# LiAl_5_O_8_:Fe^3+^ and LiAl_5_O_8_:Fe^3+^, Nd^3+^ as a New Luminescent Nanothermometer Operating in 1st Biological Optical Window

**DOI:** 10.3390/nano10020189

**Published:** 2020-01-22

**Authors:** Karolina Kniec, Marta Tikhomirov, Blazej Pozniak, Karolina Ledwa, Lukasz Marciniak

**Affiliations:** 1 Institute of Low Temperature and Structure Research, Polish Academy of Sciences, Okólna 2, 50-422 Wroclaw, Poland; k.ledwa@intibs.pl; 2 Department of Pharmacology and Toxicology, Faculty of Veterinary Medicine, Wroclaw University of Environmental and Life Sciences, ul. Norwida 25, 50-375 Wroclaw, Poland; marta.tikhomirov@upwr.edu.pl (M.T.); blazej.pozniak@upwr.edu.pl (B.P.)

**Keywords:** iron, LiAl_5_O_8_ nanocrystals, luminescent thermometry

## Abstract

New types of contactless luminescence nanothermometers, namely, LiAl_5_O_8_:Fe^3+^ and LiAl_5_O_8_:Fe^3+^, Nd^3+^ are presented for the first time, revealing the potential for applications in biological systems. The temperature-sensing capability of the nanocrystals was analyzed in wide range of temperature (−150 to 300 °C). The emission intensity of the Fe^3+^ ions is affected by the change in temperature, which induces quenching of the ^4^T_1_ (^4^G) → ^6^A_1_ (^6^S) Fe^3+^ transition situated in the 1st biological window. The highest relative sensitivity in the temperature range (0 to 50 °C) was found to be 0.82% °C (at 26 °C) for LiAl_5_O_8_: 0.05% Fe^3+^ nanoparticles that are characterized by long luminescent lifetime of 5.64 ms. In the range of low and high temperatures the S_max_ was calculated for LiAl_5_O_8_:0.5% Fe^3+^ to be 0.92% °C at −100 °C and for LiAl_5_O_8_:0.01% Fe^3+^ to be 0.79% °C at 150 °C. The cytotoxicity assessment carried out on the LiAl_5_O_8_:Fe^3+^ nanocrystals, demonstrated that they are biocompatible and may be utilized for in vivo temperature sensing. The ratiometric luminescent nanothermometer, LiAl_5_O_8_:Fe^3+^, Nd^3+^, which was used as a reference, possesses an S_max_ = 0.56%/°C at −80 °C, upon separate excitation of Fe^3+^ and Nd^3+^ ions using 266 nm and 808 nm light, respectively.

## 1. Introduction

Temperature measurement and accurate control of its value play a key role in many different fields of science, medicine and technology. In the case of bio-application, the implementation of temperature sensing may provide important information concerning metabolic processes, cell division rate and presence of inflammation. The difference in temperature of only a few degrees Celsius between healthy and cancer cells may be used as a diagnostic parameter. Additionally, during the light induced hyperthermia treatment, precise control of a specific temperature in real time enables to minimize the side effects associated with the overheating of the healthy cells. Therefore, accurate and reliable real time temperature measurement with submicron-scale spatial resolution during in vitro and in vivo experiments is of paramount importance. All these requirements are provided by luminescent thermometry, an experimental technique that enables temperature determination based on the analysis of the luminescent properties of the phosphor [1,2,3,4,5]. However, for thermometric biological applications, the phosphor employed must fulfil several requirements such as low cytotoxicity, high thermal, chemical and mechanical stability, fast response and high sensitivity to temperature changes [6,7,8]. Inorganic nanocrystals doped with optically active ions meet all these demands and thus are excellent candidates for non-contact temperature sensors. Several approaches have focused on the enhancement of the relative sensitivity of luminescent thermometers, which includes the optimization of the stoichiometry of the host material as well as the appropriate selection of the optically active ions. A current approach that is being investigated intensively relies on the utilization of the strong temperature dependent luminescence intensity of transition metal ions (TM) in comparison to the luminescence of the lanthanide ions (Ln^3+^) [9,10,11,12]. This is attributed to the considerable difference in their electronic configuration and interactions with the surrounding ligands; thus the spectroscopic features of the TM and Ln^3+^ ions differ. Because of the shielding of the 4f electrons by the 5s^2^ and 5p^6^ electrons, their interaction with the crystal field of ligands (10^2^ cm^−1^) is much less than the interaction of the d electrons with the surrounding ligands (10^4^ cm^−1^) [6,13,14]. This produces the strong electron-phonon coupling for TM ions (3d^n^ configuration) inducing a relatively large displacement (∆R) between the ground and excited states potential parabolas resulting in the intersection of the two parabolas, which facilitates nonradiative decay to the ground state. In the case of the lanthanide ions (4f^n^), the two parabolas do not intersect due to the weak electron–phonon interaction (shielding of the 4f orbitals), which results in a very small displacement (∆R ~ 0). As a consequence, nonradiative decay can only occur via multiphonon relaxation, which has a much lower probability to take place. The spectroscopic properties of some transition metal ions, Cr^3+^, Mn^2+/3+/4+^, V^3+/4+/5+^, Co^2+^, Ti^3+/4+^ [6,13,14,15,16,17,18,19,20] have been already considered for non-contact temperature sensing. However, Fe^3+^ has not been considered for this purpose.

Herein, we present for the first time the synthesis and characterization of LiAl_5_O_8_:Fe^3+^ and LiAl_5_O_8_:Fe^3+^/Nd^3+^ for application as nanocrystalline luminescent thermometers based on intensity and bandshape. The high susceptibility of Fe^3+^ ions emission intensity to temperature changes enables to develop highly sensitive nanocrystalline luminescent thermometers (LT) that can be applied in the 1st biological transparency window spectral range. The optimization process, which includes the size of the nanoparticles as well as Fe^3+^ dopant concentration, was used in order to enhance the relative sensitivity of LT in the physiological temperature range.

## 2. Materials and Methods

### 2.1. Synthesis of Fe^3+^-Doped LiAl_5_O_8_

The LiAl_5_O_8_ nanocrystals doped with Fe^3+^ ions (LiAl_5_O_8_:x% Fe^3+^) with 0.01%, 0.05%, 0.1%, 0.5%, 1% and 2% dopant concentration have been successfully synthesized by the use of the Pechini method [21]. Stoichiometric amounts of lithium carbonate (Li_2_CO_3_ of 99.999% purity from Alfa Aesar), aluminum nitrate nonahydrate (Al(NO_3_)_3_^.^9H_2_O of 99.999% purity from Alfa Aesar) and iron (III) nitrate nonahydrate (Fe(NO_3_)_3_^.^9H_2_O of 99.999% purity from Alfa Aesar) were diluted with small volume of distilled water. The mixture was heated up to 90 °C and magnetically stirred in a beaker for 2 h in an aqueous solution of citric acid (CA). The molar ratio of CA to total amount of metal was set to 2:1. This step is similar for the formation of all the metal complexes, consisting of Cit^3−^ ligands and Li^+^, Al^3+^ and Fe^3+^ central metal ions. Subsequently, an adequate volume of PEG-200 was added to the clear solution under stirring and the reaction for 1 h (PEG-200 to Cit^3−^ was 1:1). Afterwards, the mixture was transferred to a porcelain crucible and heated up to 250 °C in air to complete evaporation of water and formation of the resin. Finally, the nanocrystals were produced by annealing the resin in air for 12 h at 850 °C. Additionally, the LiAl_5_O_8_ nanocrystals doped with 0.05% of Fe^3+^ were also calcinated at 900 °C, 1000 °C and 1100 °C.

### 2.2. Synthesis of Fe^3+^, Nd^3+^ -Co-Doped LiAl_5_O_8_

LiAl_5_O_8_: Fe^3+^, Nd^3+^ nanocrystals were synthesized using the same method as presented above. The neodymium nitrate (Nd(NO_3_)_3_) was synthesized using neodymium oxide (Nd_2_O_3_ of 99.999% purity from Stanford Materials Corporation) via the dissolution of an appropriate amounts of Nd_2_O_3_ in distilled water and ultrapure nitric acid (96%) and, subsequently, triple recrystallization by the use of small volume of distilled water. The mixture of all substrates, including neodymium nitrate, was heated to produce the resin. The nanopowders were obtained by annealing in air at 850 °C for 12 h. The nominal concentration of Fe^3+^ and Nd^3+^ was 0.05% and 1% with respect to Al^3+^ and Li^+^ ions.

### 2.3. Characterization

Powder diffraction studies were carried out on PANalytical X’Pert Pro diffractometer equipped with an Anton Paar TCU 1000 N Temperature Control Unit and using Ni-filtered Cu *Kα* radiation (*V* = 40 kV, *I* = 30 mA).

Transmission electron microscope images were obtained using a TEM Philips CM-20 SuperTwin operating at 160 kV with an optical resolution of 0.25 nm.

The emission spectra were measured using the 266 nm excitation line from a laser diode (LD) and a Silver-Nova Super Range TEC Spectrometer form Stellarnet (1 nm spectral resolution). The temperature of the sample was controlled using a heating stage from Linkam (0.1 °C temperature stability and 0.1 °C set point resolution).

Luminescence decay profiles were recorded using FLS980 Fluorescence Spectrometer from Edinburgh Instruments with μFlash lamp as an excitation source and the signal was detected using a R928P side window photomultiplier tube from Hamamatsu.

### 2.4. Cytotoxicity Assessment

Cytotoxicity assessment was carried out on murine macrophage (J774.E) and fibroblast (3T3/Swiss Albino) cell lines. The choice of the in vitro model was based on the fact that under in vivo conditions, macrophages form the primary line of response to particulate matter [22,23] Thus, they are responsible for the distribution and clearance of nanoparticles and their agglomerates. On the other hand, 3T3 cells are a standard model to screen for cytotoxicity of biomaterials as fibroblasts are the main cellular component of connective tissues [24,25]. Cells were cultured in RPMI-1640 medium (Institute of Immunology and Experimental Therapy, Wroclaw, Poland) supplemented with 10% fetal bovine serum (FBS, Sigma, St. Louis, MO, USA), L-glutamine (Sigma, Welwyn Garden City, UK) and antibiotic (streptomycin and penicillin, Sigma, Munich, Germany). For the cytotoxicity assessment, cells were seeded in 96-well-plates (TTP, Basel, Switzerland) at a density of 3 × 10^3^ (3T3) or 10 × 10^3^ (J774.E) cells per well and pre-incubated at 37 °C for 24 h in a humidified atmosphere of 5% CO_2_. Afterwards, nanoparticle dispersions were added. Stock dispersions were prepared based on a simplified version of the NANOGENOTOX dispersion protocol. Nanoparticles were suspended in 0.05% BSA water solution and bath-sonicated at room temperature for up to 5 min. The stock solutions were further diluted in 0.05% BSA and dispersions in complete culture medium were prepared. In parallel, the highest nanoparticle concentrations were centrifuged at 30,000× *g* for 3 h, and the particle-free supernatants were used as a diluent control (to exclude any possible particle-unrelated effects due to the presence of soluble compounds). Cells were exposed to the dispersions for 48 h (5% CO_2_, 37 °C). Subsequently, the MTT assay was carried out. The test is based on the enzymatic reduction of the tetrazolium salt MTT [3-(4,5-dimethylthiazol-2-yl)-2,5-diphenyl-tetrazoliumbromide] in living, metabolically active cells. The metabolite, purple-colored formazan is measured colorimetrically, using a multiwell plate reader. Preliminary experiment showed no interference of the nanoparticles with MTT or formazan in a cell-free system at concentrations of 50 µg/mL and lower. Thus, 50 µg/mL was chosen as the highest concentration in this study. After 4 h of incubation, cells were lysed and the optical density (OD) was measured after 24 h using a spectrophotometric microplate reader (Tecan Spark 10M, Männedorf, Switzerland) at a wavelength of 570 nm (reference 630 nm). The OD of control cells was taken as 100%. Cell viability was determined as follows: % viability = (mean OD in the test wells/mean OD for control wells) × 100. The results were obtained from at least 3 independent experiments.

## 3. Results

**Figure 1:**
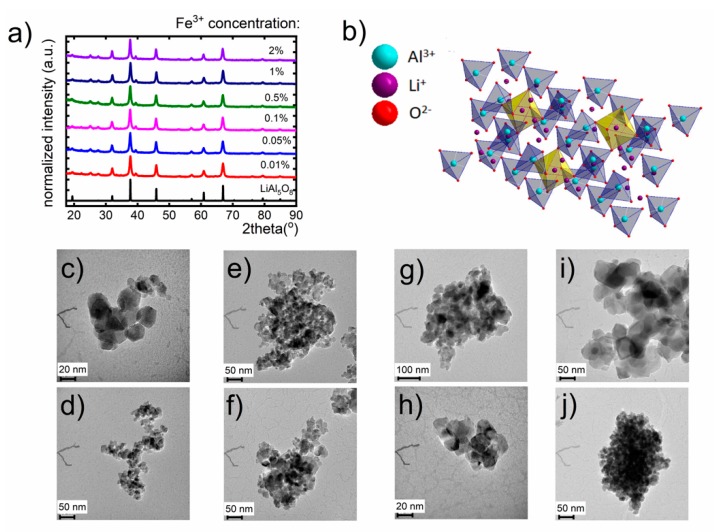
(**a**) XRPD patterns of LiAl_5_O_8_:Fe^3+^ nanocrystals of different dopant concentration; (**b**) the visualization of LiAl_5_O_8_ structure; respective TEM images for LiAl_5_O_8_ nanocrystals annealed at (**c**,**d**), 850 °C, (**e**,**f**) 900 °C, (**g**,**h**) 1000 °C and (**i**,**j**) 1100 °C, respectively.

X-ray powder diffraction (XRPD) was used to establish the crystalline structure and phase purity of the LiAl_5_O_8_:Fe^3+^ nanocrystals with different dopant concentration and annealed at different temperatures (Figure S1). In Figure 1a, the XRPD experimental pattern of LiAl_5_O_8_:Fe^3+^ nanocrystals is shown. The presence of the principal diffraction peaks as well as the other diffraction peaks are in agreement with the reported pattern for LiAl_5_O_8_ (ICSD File No. 10480). This confirms the presence of cubic LiAl_5_O_8_ crystallizing with space group P4_3_32. The Li^+^ and Al^3+^ ions are 6-fold coordinated (LiO_6_)^11−^ and (AlO_6_)^9−^ (B-site) while the other Al^3+^ ions are four coordinated tetrahedra (AlO_4_)^5−^ (A-site) [26,27,28]. The ratio of the octahedral sites occupied by Li^+^ and Al^3+^ ions is 1:3 (Figure 1b) [26,29]. It is worth noting that above 1295 °C the ordered LiAl_5_O_8_ structure is transformed into disordered form [26,27,30]. The broadening of XRPD peaks results from the small size of the nanoparticles. The shift of the XRPD peaks with respect to the reference pattern observed for higher concentration is related with the increase of the unit cell caused by the difference in the ionic radii between Fe^3+^ and Al^3+^, which is reflected in the increase of cell parameters from 7.9103 Å for 0.1% Fe^3+^ to 7.9177 Å for 2% Fe^3+^. Moreover, slight enlargement of the average grain size from 16.21 nm up to 20.39 nm with dopant concentration was found, which results from the difference in the ionic radii between dopant and the substituted ions (Fe(III) = 63 pm and Al (III) = 53 pm) (Figure S2). The Nd^3+^ ions occupy the Li^+^ site, whereas Fe^3+^ ions may substitute both in the A-sites and B-sites for Al^3+^ ions due to the similarities of ionic radii (0.69 Å, 0.675 Å for 6-fold and 0.63 Å, 0.53 Å for 4-fold coordinated Fe^3+^ and Al^3+^ ions, respectively). However, as it has been previously shown, the Fe^3+^ ions occupy mainly A-sites, forming the FeO_4_^5−^ clusters in the LiAl_5_O_8_ structure [26,27,28,29,31,32]. The representative TEM images (Figure 1c–j) depict that LiAl_5_O_8_:Fe^3+^ material comprise well-crystalized and agglomerated grains. The analysis of the impact of the annealing temperature on the structure of LiAl_5_O_8_ nanocrystals shows that the average grain size increases from 16.5 nm to 40 nm as the temperature increases from 850 °C to 1100 °C.

**Figure 2:**
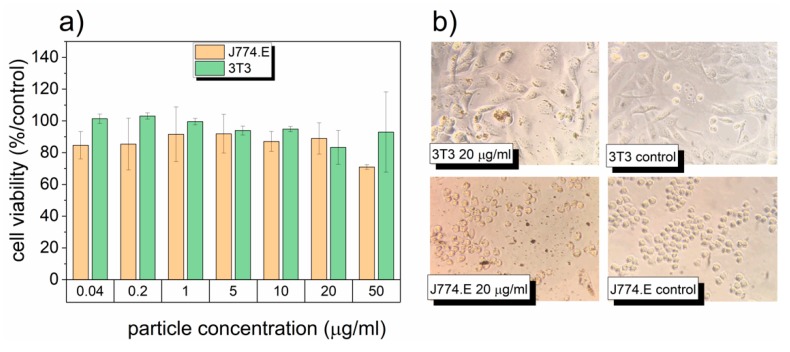
(**a**) Mean (±SD) viability of J774.E macrophages and 3T3 fibroblasts exposed for 48 h to different concentration of LiAl_5_O_8_:Fe^3+^. Viability determined by MTT assay and expressed as the percent of control; (**b**) 3T3 and J774.E cells exposed to LiAl_5_O_8_:Fe^3+^ nanoparticles in concentration 20 μg/mL and their corresponding controls (magnification 400×).

**Figure 3:**
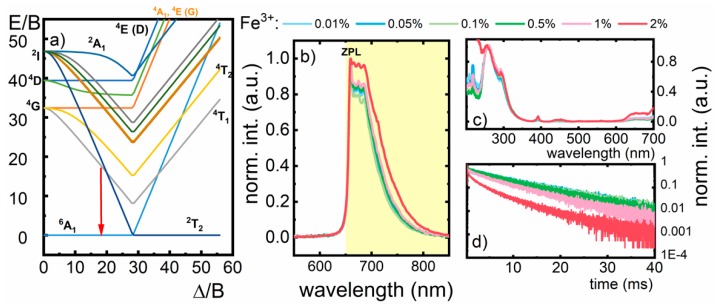
(**a**) Tanabe–Sugano diagram of d^5^ electronic configuration of Fe^3+^ ions; (**b**) the emission spectra of LiAl_5_O_8_:Fe^3+^ nanocrystals measured upon λ_exc_ = 266 nm; (**c**) excitation spectra of Fe^3+^ ions for λ_em_ = 720 nm; (**d**) decay profile of the ^4^T_1_ (^4^G) → ^6^A_1_ (^6^S) emission for 720 nm.

In order to verify the potential use of LiAl_5_O_8_:Fe^3+^ nanoparticles for biomedical applications, cytotoxicity assessment was carried out using J774.E and 3T3 cell lines. The effects of LiAl_5_O_8_:Fe^3+^ on cell viability are summarized in Figure 2. The LiAl_5_O_8_:Fe^3+^ nanoparticles exhibit no significant effect on cell viability, at the highest concentration of 50 µg/mL. In our experiment, J774.E macrophages appeared to be slightly less viable when exposed to the highest concentration, whereas the response of fibroblasts was found to be much more variable. This may be related to the instability of the dispersion. Lower stability translates into higher dose delivered directly to cells over time, and the actual exposure is quite complicated to predict [33]. Notwithstanding this lack of stability, LiAl_5_O_8_:Fe^3+^ appears to be non-toxic in both cellular models. Due to negligible toxicity, it was not possible to calculate the half maximal inhibitory concentration, the typical parameter used to characterize the toxic potency of substances. Figure 3 shows the effects on cell morphology and partial cellular uptake of LiAl_5_O_8_:Fe^3+^ by both cell types as determined by light microscopy. Although J774.E (in contrast to 3T3) are known to be efficient phagocytes [34], the uptake of LiAl_5_O_8_:Fe^3+^ was low in both cell lines. In conclusion, LiAl_5_O_8_:Fe^3+^ were found to be biocompatible in the applied in vitro model and may be considered for further biological investigation.

In order to understand the luminescent properties of the Fe^3+^ ions, the Tanabe–Sugano diagram for d^5^ electronic configuration should be considered (Figure 3a). According to the previously reported studies, the crystal field strength for LiAl_5_O_8_:Fe^3+^ is Dq = 800 cm^−1^, which corresponds to a Δ/B ~ 18.1. In this case, the luminescence of the Fe^3+^ ions should be due to the ^4^T_1_ (^4^G) → ^6^A_1_ (^6^S) electronic transition [35]. In agreement with our prediction upon 266 nm excitation at −150 °C, the LiAl_5_O_8_:Fe^3+^ nanocrystals reveal deep red emission centered at 660 nm (Figure 3b). The observed sharp peak in the emission band is attributed to the zero-phonon line (ZPL) ^4^T_1_ (^4^G) → ^6^A_1_ (^6^S) *d-d* electronic transition of Fe^3+^ ion (Figure 3b). It should be noted that the intensity of the ZPL decreases with dopant concentration, which may be attributed to the interionic Fe^3+^-Fe^3+^ interaction. At a Fe^3+^ concentration of 2%, the ZPL is barely observable. The excitation spectrum of LiAl_5_O_8_:Fe^3+^, recorded at 650 nm (^6^A_1_ (^6^S) → ^4^T_1_ (^4^G) transition), consists of the broad band in UV spectral region (200–300 nm), which is related to the O^2−^ → Fe^3+^ charge transfer (CT) transition and two less intensive absorption bands at 390 nm and 440 nm attributed to the ^6^A_1_ (^6^S)→^4^A_1_ (^4^G), ^4^E (^4^G) and ^6^A_1_ (^6^S) →^4^T_2_ (^4^G) *d-d* electronic transitions, respectively (Figure 3d; see also Figure S3). No significant difference is observed in the excitation spectra for different Fe^3+^ concentration. However, slight increase in the intensity of the *d-d* absorption bands with respect to the CT band was observed as a function of Fe^3+^ concentration. Due to its significantly higher absorption cross section with respect to the *d-d* bands, the CT absorption band was used for excitation. Additionally, with the increase of the nanoparticle size, a gradual enhancement of the absorption band, attributed to the ^6^A_1_ (^6^S) → ^4^T_1_ (^4^G) electronic transition, was observed (see Figure S6). Therefore, considering the information obtained from the analysis of the excitation spectra, the following mechanism of the Fe^3+^ luminescence from the A-site can be proposed: Upon 266 nm excitation, the electron transfer from O^2−^ to the Fe^3+^ takes place reducing it to Fe^2+^ followed by the transfer of the energy to the excited states of Fe^3+^ ions. The radiative depopulation of ^4^T_1_ (^4^G) excited state to ^6^A_1_ (^6^S) ground state leads to the generation of the emission band at 660 nm. It was found that dopant concentration influenced both the luminescent lifetimes of Fe^3+^ excited state and the shape of the decay profile (Figure 3d). Nanocrystals doped with 0.05% Fe^3+^ showed exponential decay profiles and lifetime as long as 5.64 ms. However, when the concentration of the dopant was increased, the decay profiles were non-exponential and the lifetimes were significantly shorter, 1.96 ms for 2% Fe^3+^ (average lifetime). On the other hand, no significant influence of the size of the nanoparticle on the lifetime was found, which confirms that surface-related nonradiative depopulation processes have a minor impact on the kinetics of the ^4^T_1_ (^4^G) state (see Figure S5).

**Figure 4:**
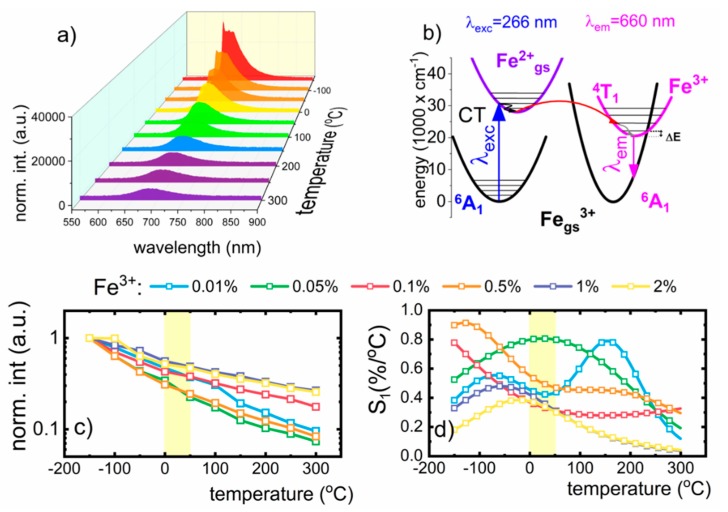
(**a**) Thermal evolution of emission spectra of LiAl_5_O_8_:0.05%Fe^3+^ nanocrystals upon λ_exc_ = 266 nm; (**b**) the schematic configurational coordinates diagram of Fe^3+^ ions energy levels; (**c**) the temperature impact on the emission intensity of Fe^3+^ ions annealed at 850 °C; (**d**) corresponding relative sensitivities based on their thermally-affected luminescence.

Spectroscopic properties of Fe^3+^ ions in LiAl_5_O_8_ nanocrystals were analyzed in terms of their potential use for non-contact temperature sensing applications, and therefore, their emission spectra were analyzed in a wide range of temperatures, −150 °C to 300 °C (Figure 4a; see also Figure S4). It was observed that the emission from the Fe^3+^ ion is highly susceptible to luminescence thermal quenching, and the increase in temperature causes the reduction of the intensity of 660 nm emission band in the temperature range studied. At 300 °C, the emission from the ^4^T_1_ (^4^G) → ^6^A_1_ (^6^S) transition was very weak, and the shape of the emission bands was affected by temperature. The intensity of ZPL is quenched at low temperatures, and above −50 °C, only a broad emission band was observed. Figure 4b shows the configuration coordinate diagram, which can be used to explain the mechanism of the luminescence thermal quenching of the Fe^3+^ ions. The displacement of the excited ^4^T_1_ (^4^G) state parabola with respect to the ground ^6^A_1_ (^6^S) state results in the intersection of the two parabolas (Figure 4b). The energy difference ΔE between the lowest point of the excited state parabola and the intersection point represents the energy barrier for the electron to overcome such that nonradiative decay occurs. The energy difference for the LiAl_5_O_8_:Fe^3+^ nanocrystals was calculated, and a value of 585 cm^−1^ was obtained. This barrier can be overcome by relatively small thermal energy resulting in nonradiative decay. The rate of thermal quenching of the Fe^3+^ luminescence is dependent on dopant concentration, and it decreases at higher Fe^3+^ concentration. Because the spectral positions of both the absorption and emission bands are independent of dopant concentration, this difference is not related with the change of the activation energy, ΔE (see Figure S7). Therefore, the dominant process responsible for the concentration effect on luminescence thermal quenching is energy diffusion to the defects. The probability of energy diffusion among excited states of Fe^3+^ enhances with shortening of the average distance between interacting ions, resulting from the higher dopant concentration. In the case of the nanosized phosphor, distance between emitting center and the nanoparticle surface defects is relatively low facilitating this kind of interaction. Additionally, the difference in the ionic radii between dopant ions and substituted host ions may lead to the formation of local point defects at high dopant concentration, which increases the probability of energy transfer to the defects. The same effect is responsible for luminescence concentration quenching in TM doped phosphors. Similar observation has been reported for Mn^3+^ doped YAG nanocrystals [18]. This concentration quenching leads to the lowering of the overall emission intensity observed for higher dopant concentration. Therefore, the efficiency of the additional quenching of luminescence induced by the increase of temperature is minimized. It is worth noting here that at a temperature up to 300 °C the complete luminescence quenching is not observed for the LiAl_5_O_8_:Fe^3+^ nanocrystals, which improves the accuracy of non-contact temperature readout due to high signal-to-noise ratio even at high temperatures (Figure 4a and Figure S4). To quantify observed luminescent response of the Fe^3+^ doped nanocrystals on temperature changes, the relative sensitivity of luminescence nanothermometers was calculated according to the following equation:

(1) $$S_{1}=\frac{1}{x}\frac{\Delta x}{\Delta T}\cdot 100\%$$

where *x* refers to the temperature dependent parameter, which in this case is represented by emission intensity, and Δ*x* represents the change of x for ΔT change of temperature. The results of S_1_ as a function of temperature for different dopant concentrations are presented in Figure 4d. As it can be noted, thermal evolution of S_1_ strongly depends on dopant concentration. Based on the value obtained, S_1_ = 0.82%/°C, for the LiAl_5_O_8_:0.05% Fe^3+^ nanocrystals, it can be concluded that the most promising performance for non-contact temperature sensing is in the range 20–50 °C. At low temperature, a value of 0.92%/°C for S_1_ was obtained at −100°C.

**Figure 5:**
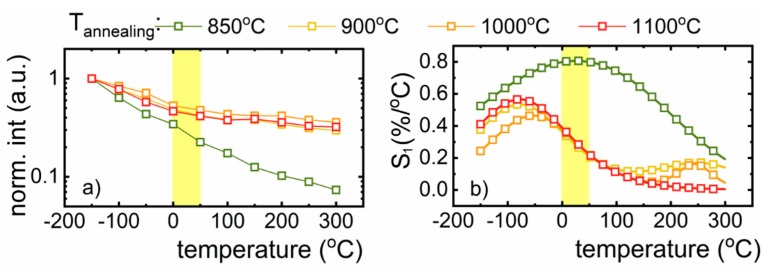
(**a**) The temperature impact on the emission intensity of 0.05% Fe^3+^ ions annealed at different temperatures; (**b**) corresponding relative sensitivities based on their thermally affected luminescence.

Taking into account the excellent performance for temperature sensing at a concentration of 0.05% Fe^3+^ doped nanocrystals, this concentration was chosen for further investigations of the size of the nanoparticles and its correlation to the relative sensitivity of nanothermometer. The rate of luminescence thermal quenching decreases with the increase in size of the nanoparticle’s (Figure 5a). As it has already been shown in the case of the nanoparticles with an average grain size 16.5 nm, the integrated emission intensity decreases by one order of magnitude in −150 to 300 °C temperature range, while for nanoparticles of 18, 30 and 40 nm size, the emission intensity decreases to 29%, 32% and 36% of its initial (at −150 °C) value, respectively. This phenomenon is probably due to the number of the ions located on the surface of the nanocrystals, which are more susceptible to luminescence thermal quenching with respect to those located in the bulk part of the nanoparticle. By increasing the grain size, the number of surface ions reduces with respect to the total number of Fe^3+^ ions, and hence, their impact on the luminescent properties of the nanocrystals is less evident. Similar observation has been already reported for the NaYF_4_:Yb^3+^,Er^3+^ nanocrystals [36]. This behavior is reflected in the values of the relative sensitivities presented in Figure 5b, which reveal a single maxima for each of the sizes of the nanoparticles in the temperature range under investigation. The value of the maximal relative sensitivity and the temperature at which this S_max_ occurs depend on the size of the nanoparticles as follows: S_max_ = 0.82%/°C (at 26 °C) for nanoparticles of 16.5 nm size, 0.57%/°C (at −83 °C) for 18 nm, 0.44%/°C (at −60 °C) for 30 nm and 0.53%/°C (at −83 °C) for 40 nm (Figure 5b).

**Figure 6:**
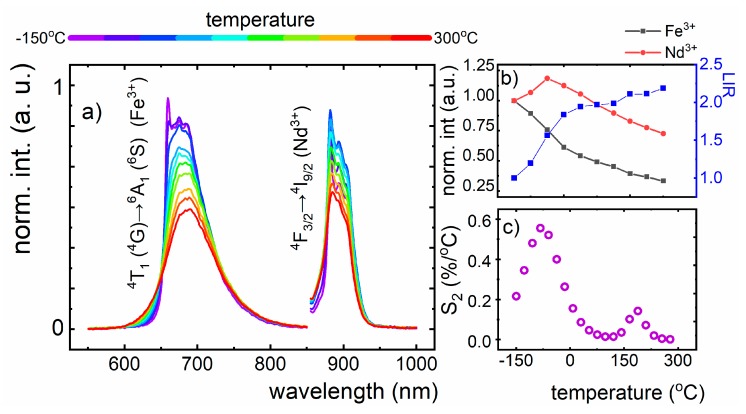
(**a**) Thermal evolution of emission spectra of Fe^3+^ and Nd^3+^ ions in LiAl_5_O_8_ nanocrystals, upon 266 nm and 808 nm excitation, respectively; (**b**) the emission intensity behavior of LiAl_5_O_8_:Fe^3+^, Nd^3+^ nanocrystals under the temperature increment; (**c**) the relative sensitivity of LiAl_5_O_8_:Fe^3+^, Nd^3+^- based luminescence nanothermometers.

The maximum emission intensity of Fe^3+^ in LiAl_5_O_8_, centered at 660 nm, overlaps with the 1st biological optical window, which is beneficial in terms of temperature sensing of both living human and animal cells, significantly increasing its applicability in biological and medicinal field. Taking advantage of the fact, that the emission intensity of lanthanide ions (Ln^3+^) is influenced much less by changes in the environment in comparison to transition metal ions (TM), we designed a new LiAl_5_O_8_:Fe^3+^, Nd^3+^ luminescence nanothermometers, based on the thermal susceptibility of the Fe^3+^ emission and using Nd^3+^ as the reference signal. The Nd^3+^ ions were introduced as a co-dopant, because the ions do not show spectral overlap (emission) with Nd^3+^. It was found that 266 nm excitation does not provide the simultaneous emission of Fe^3+^ and Nd^3+^ ions. The investigation on the susceptibility of Fe^3+^, Nd^3+^- based thermometer to the temperature changes were carried out in the range of −150 to 300 °C (Figure 6a). The ^4^T_1_ (^4^G) → ^6^A_1_ (^6^S) emission of Fe^3+^, centered at 660 nm, was recorded using 266 nm irradiation, enabling the observation of ZPL transition. In turn, the emission spectrum of Nd^3+^ ions was measured upon 808 nm excitation, providing emission with the maximum at 880 nm, which is attributed to the ^4^F_3/2_ → ^4^I_9/2_ transition. What is more, this excitation wavelength does not induce the population of Fe^3+^ energy states, from which the emission occurs. The temperature enhancement leads to the reduction of emission intensity of both *d-d* and *f-f*; however, the Fe^3+^ luminescence is affected to a greater extent (Figure 6b). In the case of the Nd^3+^ ions, this phenomenon is observed above −50 °C. Since the emission intensities of Fe^3+^ and Nd^3+^ ions are thermally dependent, their potential performance to temperature non-contact readout was taken into account. To this aim, the luminescent intensity ratio (LIR) was defined, using the below equitation:

(2) $$LIR=\frac{Fe^{3+}({\;}^{4}T_{1}({\;}^{4}G)\to {\;}^{6}A_{1}({\;}^{6}S))}{Nd^{3+}({\;}^{4}F_{3/2}\to {\;}^{4}I_{9/2})}=\frac{ʃI660nm}{ʃI880nm}$$

As the temperature increases, the LIR value rises; however, the most evident growth was found for low temperatures, namely from −150 to 0 °C (Figure 6b). The relationship between luminescent intensity and temperature changes allows the calculation of the relative sensitivity, quantitatively showing the predisposition of LiAl_5_O_8_:Fe^3+^, Nd^3+^ nanocrystals for temperature sensing, and is calculated as follows:

(3) $$S_{2}=\frac{1}{LIR}\frac{\Delta LIR}{\Delta T}\cdot 100\%$$

The maximum value of the S_2_ was found to be 0.56 %/°C at −80 °C, and it decreases along with the temperature increase up to 0.013%/°C at 100 °C. It rises and reaches the value of 0.15%/°C at 185 °C. In terms of potential biological application of LiAl_5_O_8_:Fe^3+^, Nd^3+^ luminescence nanothermometers, the maximum S_2_ was calculated to be 0.18%/°C at 0 °C (Figure 6c).

In turn, for other Fe^3+^ ions concentrations, the highest relative sensitivities, despite being considerable, fall on different temperature regions, and none of them overlap with the physiological temperature range (Figure 5b). Moreover, in case of higher temperature sensing, which is important primarily for other application such as controlling of local overheating of parts of mechanical and electronic devices, the LiAl_5_O_8_:0.01%Fe^3+^ nanothermometer (S_1max_ = 0.79%/°C at 150 °C) (Figure 4d) would be the most appropriate.

## 4. Conclusions

The LiAl_5_O_8_:Fe^3+^, LiAl_5_O_8_:Fe^3+^: Nd^3+^ nanomaterials were successfully synthesized via modified Pechini method, taking advantage of resin formation within few hours, in which Fe^3+^ occupy the octahedral sites of Al^3+^. The emission spectrum of LiAl_5_O_8_:Fe^3+^ nanocrystals consists of a strong emission band, centered at 660 nm, overlapping with the 1st biological optical window, which significantly increases their biomedical applicability. The emission band can be attributed to the ^4^T_1_ (^4^G) → ^6^A_1_ (^6^S) *d-d* electronic transition, while the sharp peak is attributed to the zero-phonon line (ZPL), which deceases in intensity as the concentration of Fe^3+^increases. Above −50 °C the ZPL is quenched and only a broad emission band is observed. The increase in concentration of Fe^3+^ leads also to the shortening of luminescent lifetime from 5.64 ms (0.05% of Fe^3+^) to 1.96 ms (2% of Fe^3+^), which is related to the Fe^3+^-Fe^3+^ interaction, causing the non-exponential decay and the slight enlargement of the average grain size form 16.21 nm to 20.39 nm, respectively. In turn, the increase of the annealing temperature causes the grain size to grow up to 40 nm at a calcination temperature of 1100 °C. The emission intensity of Fe^3+^ ions is steadily quenched in the temperature range investigated (−150 to 300 °C). However, at 300 °C, the Fe^3+^ luminescence is not totally reduced, which provides the lowering of signal-to-noise ratio and thus improvement of sensing accuracy even at very high temperatures, where S_max_ value was calculated to be 0.79%/°C at 150 °C for LiAl_5_O_8_:0.01% Fe^3+^. It was found that in the range of biological temperatures, the most susceptible emission intensity to the environment changes was for a nanoparticle size of 16.5 nm (LiAl_5_O_8_:0.05% Fe^3+^), reaching the maximal value of relative sensitivity of 0.82%/°C at 26 °C, thus being promising for biological applications. The biocompatibility of Fe^3+^- doped luminescent thermometers for in-vitro sensing was confirmed by cytotoxicity investigations. Possible applications in in vivo conditions require further investigations. Since the emission intensity of the Nd^3+^ ion is affected to a lesser extent by temperature in comparison to the Fe^3+^, ion, the ratiometric nanothermometer was developed, using Nd^3+^ luminescence as reference signal, which has a S_max_ value of 0.56%/°C at −80 °C. However, to detect the thermally influenced luminescence of LiAl_5_O_8_:Fe^3+^: Nd^3+^ thermometer, nanocrystals must be exposed to two excitation wavelengths, namely 266 nm and 808 nm, consequently enabling the emission transitions of Fe^3+^ and Nd^3+^ ions. To sum up, the luminescent thermometer based on the Fe^3+^ emission intensity can be considered as a promising material for non-contact temperature readout in a wide range of applications.

## Supplementary Materials

The following are available online at https://www.mdpi.com/2079-4991/10/2/189/s1. Figure S1, XRD patterns of LiAl_5_O_8_:0.05% Fe^3+^ nanocrystals annealed at different temperatures; Figure S2, the influence of Fe^3+^ dopant concentration of a cell parameter and grain size; Figure S3, excitation spectrum for LiAl_5_O_8_:0.05%Fe^3+^ nanocrystals annealed at different temperatures, for λ_em_ = 720 nm; Figure S4, luminescence life time for 660 nm and 720 nm emission intensity, corresponding to the ^4^T_1_ → ^6^A_1_ transition at λ_em_ = 266 nm; Figure S5, luminescence lifetime for LiAl_5_O_8_:0.05%Fe^3+^ nanocrystals annealed at different temperatures, for λ_em_ = 720 nm; Figure S6, the emission intensity of LiAl_5_O_8_:Fe^3+^ nanocrystals with different dopant concentration, annealed at 850 °C and recorded under 266 nm excitation; Figure S7, activation energy defined for LiAl_5_O_8_ nanocrystals, annealed at 850 °C, doped with different Fe^3+^ concentration.
