# Supplementary material for: LiAl5O8:Fe3+ and LiAl5O8:Fe3+, Nd3+ as a New Luminescent Nanothermometer Operating in 1st Biological Optical Window
Source: Nanomaterials (Basel). 2020 Jan 22;10(2):189. doi: 10.3390/nano10020189 (PMC7074848; doi:10.3390/nano10020189)
Supplement: Supplementary file 1 [file nanomaterials-10-00189-s001.pdf]

SUPPORTING INFORMATION

$LiAl_5O_8:Fe^{3+}$ and $LiAl_5O_8:Fe^{3+}$, Nd^{3+} as a new luminescent nanothermometer operating in 1st biological optical window

Karolina Kniec*[1] , Marta Tikhomirov [2] , Blazej Pozniak [2] , Karolina Ledwa [1] , Lukasz Marciniak *[1]

[1] Institute of Low Temperature and Structure Research, Polish Academy of Sciences, Okólna 2, 50-422 Wroclaw, Poland; k.ledwa@intibs.pl

[2] Department of Pharmacology and Toxicology, Faculty of Veterinary Medicine, Wroclaw University of Environmental and Life Sciences, ul. Norwida 25, 50-375 Wroclaw, Poland; marta.tikhomirov@upwr.edu.pl (M.T.); blazej.pozniak@upwr.edu.pl (B.P.)

* Correspondence: k.kniec@intibs.pl (K.K.); l.marciniak@intibs.pl (L.M.)

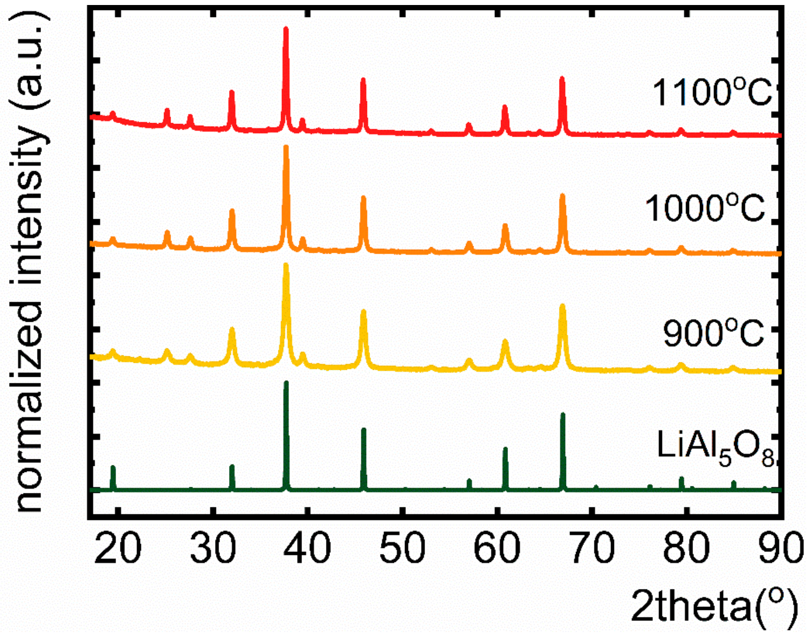

Figure S1. XRD patterns of $LiAl_5O_8$:0.05% Fe^{3+} nanocrystals annealed at different temperatures.

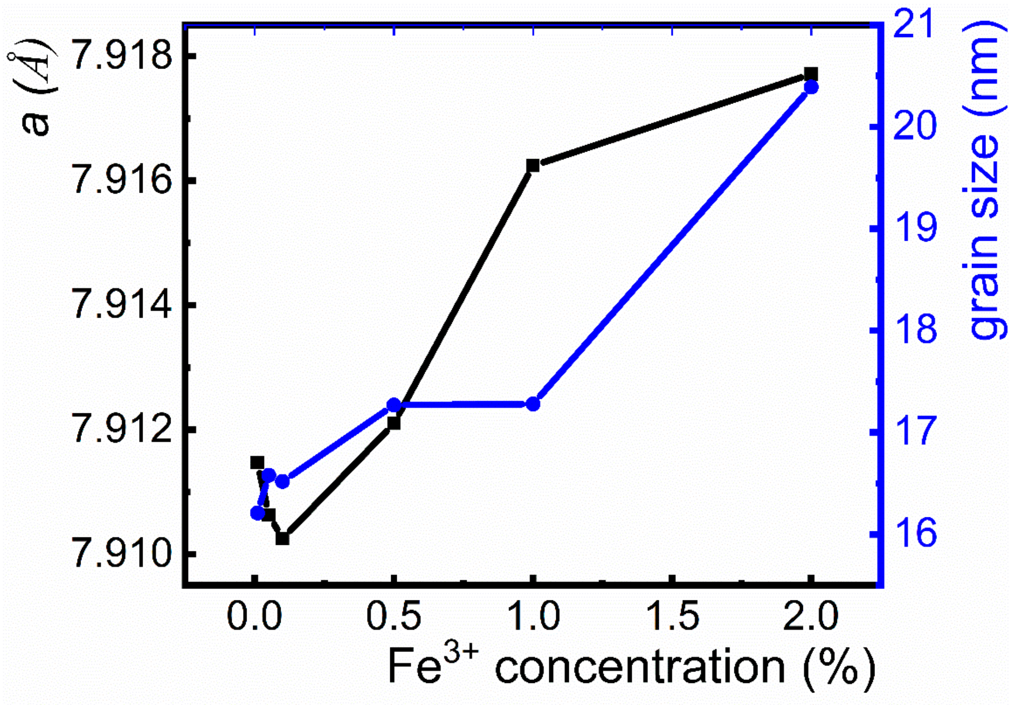

Figure S2. The influence of Fe^{3+} dopant concentration of a cell parameter and grain size.

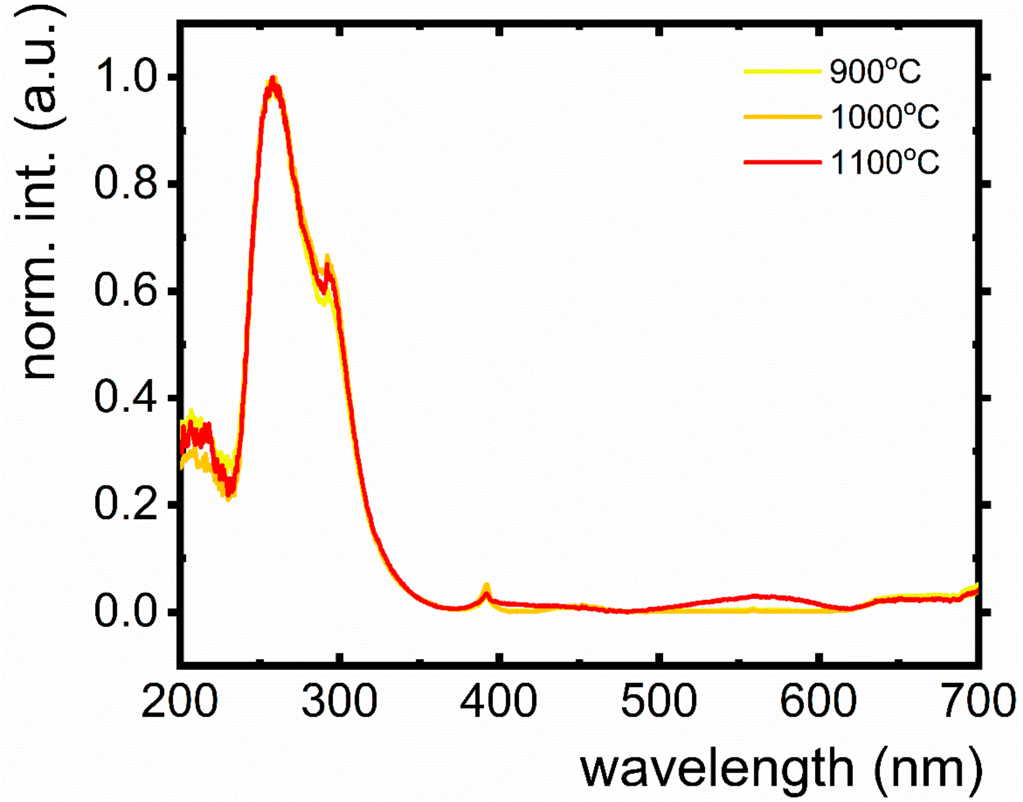

Figure S3. Excitation spectrum for $LiAl_5O_8$:0.05%Fe^{3+} nanocrystals annealed at different temperatures, for λ_{em}=720 nm.

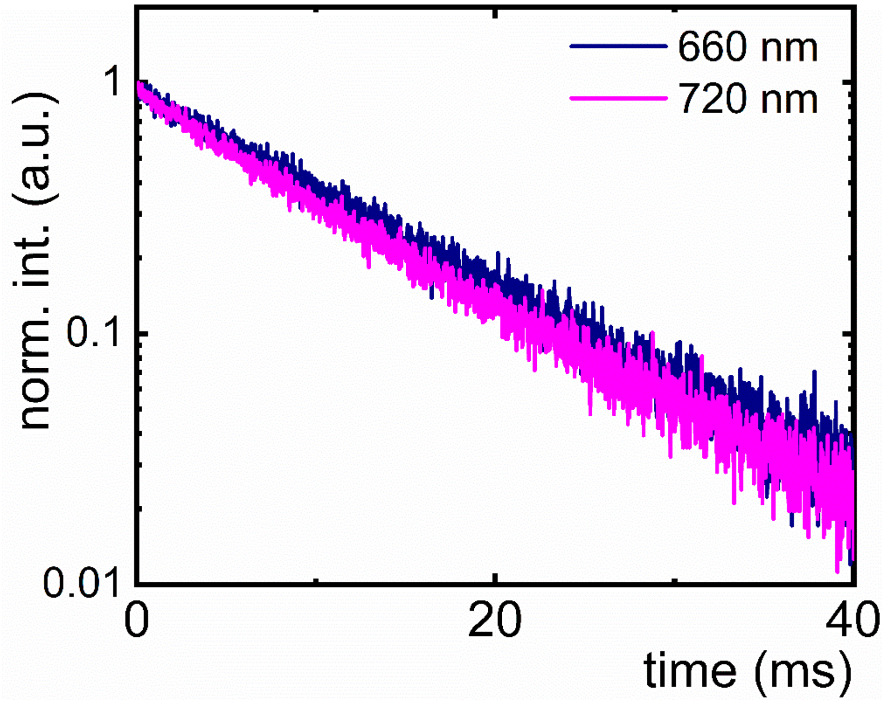

Figure S4. Luminescence life time for 660 nm and 720 nm emission intensity, corresponding to the $^4T_1 \rightarrow {}^6A_1$ transition at λ_{em}=266 nm.

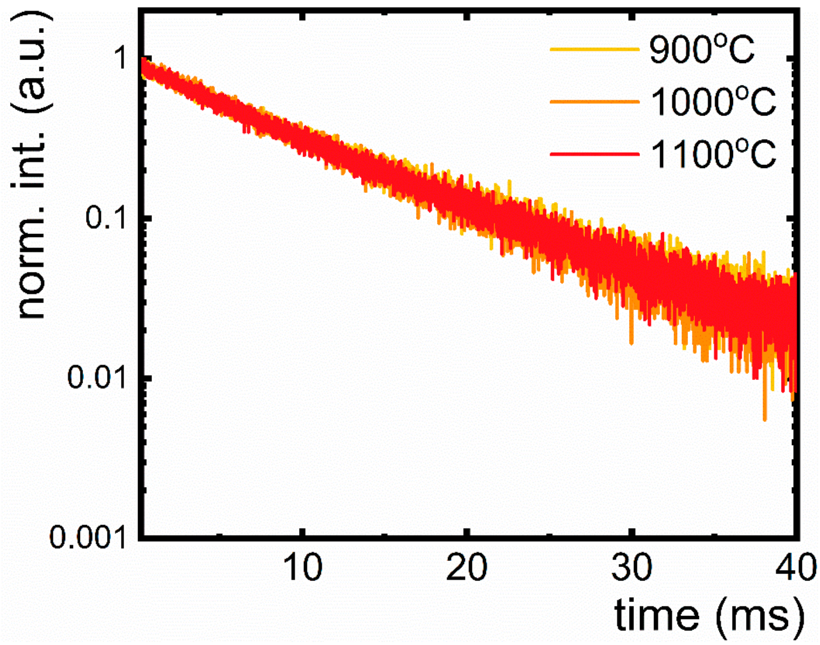

Figure S5. Luminescence lifetime for $LiAl_5O_8$:0.05%Fe^{3+} nanocrystals annealed at different temperatures, for λ_{em}=720 nm.

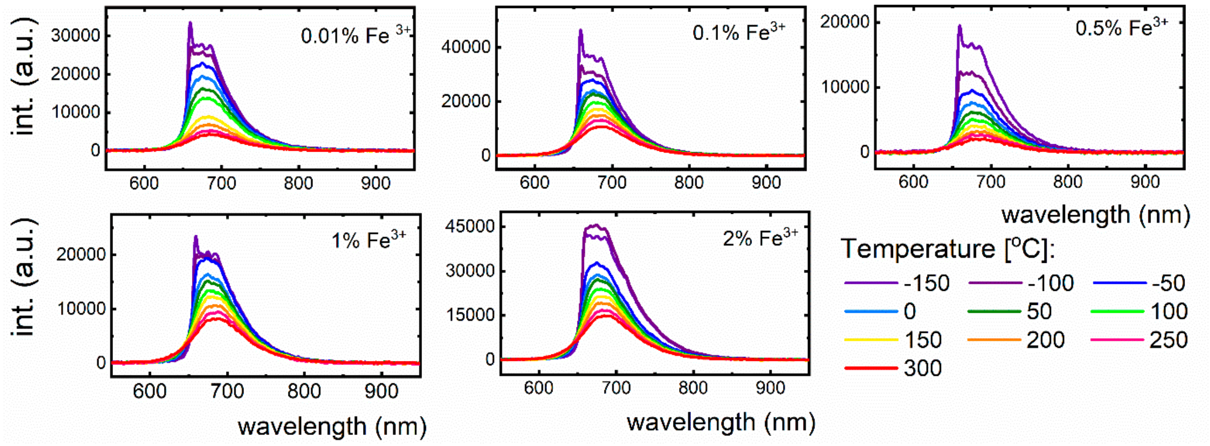

Figure S6. The emission intensity of $LiAl_5O_8:Fe^{3+}$ nanocrystals with different dopant concentration, annealed at 850°C and recorded under 266 nm excitation.

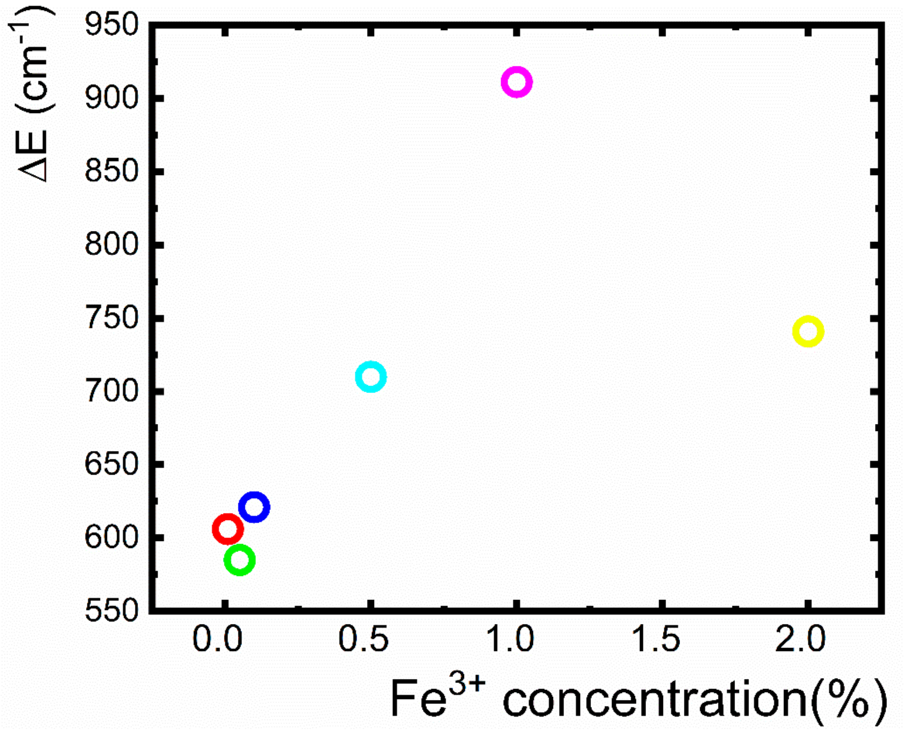

Figure S7. Activation energy defined for $LiAl_5O_8$ nanocrystals, annealed at 850°C, doped with different Fe^{3+} concentration.
